# Effects of a Flavonoid-Rich Extract from *Citrus sinensis* Juice on a Diet-Induced Obese Zebrafish

**DOI:** 10.3390/ijms20205116

**Published:** 2019-10-15

**Authors:** Giuseppe Montalbano, Manuela Mania, Maria Cristina Guerrera, Rosaria Laurà, Francesco Abbate, Maria Levanti, Alessandro Maugeri, Antonino Germanà, Michele Navarra

**Affiliations:** 1 Zebrafish Neuromorphology Lab, Department of Veterinary Sciences, University of Messina, 98122 Messina, Italy; gmontalbano@unime.it (G.M.); manuela.mania87@gmail.com (M.M.); mcguerrera@unime.it (M.C.G.); laurar@unime.it (R.L.); abbatef@unime.it (F.A.); mblevanti@unime.it (M.L.); 2 Department of Chemical, Biological, Pharmaceutical and Environmental Sciences, University of Messina, 98122 Messina, Italy; amaugeri@unime.it (A.M.); mnavarra@unime.it (M.N.)

**Keywords:** *Citrus sinensis*, flavonoids, zebrafish, diet-induced obesity, orange juice, lipolysis

## Abstract

Background: Obesity is a pathological condition that has reached epidemic proportions; hence, it is necessary to find novel strategies aimed at fighting this disease. The present study was designed to evaluate the effect of a flavonoid-rich extract of orange (*Citrus sinensis*) juice (OJe) in diet-induced obese zebrafish. Methods: Adult zebrafish were divided into four diet groups: (i) normally fed (NF); (ii) overfed (OF); (iii) NF supplemented with OJe (5 mL/L in fish water; NF + OJe); and (iv) OF supplemented with OJe (OF + OJe). Each week, body weight (BW) and body mass index (BMI) were measured, and, at the end of the fifth week, euthanized zebrafish were processed for both microscopic evaluations and qPCR analyses. Results: In OF zebrafish, OJe significantly decreased both BW and BMI values and lowered the visceral adipose tissue, while it had little effect in the NF group. Moreover, it significantly reduced adipocyte cell size in both NF and OF groups in both visceral and subcutaneous adipose tissues, as well as their number in OF fish. Finally, OJe modulated some obesity-related genes, such as leptin A, ghrelin, orexin, pro-opiomelanocortin (POMC), and neuropeptide Y (NPY), in both gut and brain. Conclusion: This study adds new insights into the anti-obesity properties of orange juice and its flavonoids, suggesting their role as weight management agents through a lipolytic action linked to a restoration of metabolism-regulating gene expression.

## 1. Introduction 

Obesity is a complex multifaceted disorder that is primarily due to an imbalance between energy intake and its expenditure, characterized by enhanced fat storage in adipose tissue and insulin-responsive organs, such as skeletal muscle and liver [1]. It is now reaching epidemic proportions and widening at an alarming rate, particularly in the Occident. Worldwide, obesity has nearly doubled since 1980. In 2016, more than 1.9 billion adults aged 18 years or over were overweight, and of these, more than 650 million were obese, corresponding to about 13% of the world adult population [2]. Obesity and its treatments have been studied in several animal models, and in this field, zebrafish have been increasingly used to explore common chronic human metabolic diseases such as obesity [3,4]. This is because they share structural and functional similarities with humans [5,6] and possess well-conserved neural and endocrine signals regulating both appetite and food intake, thus mimicking the characteristics of human diet-induced obesity [7]. Indeed, zebrafish respond very well to food stimulus (freshly hatched *Artemia* nauplii) and, in only four weeks, they develop obesity, sharing the same pathophysiological conditions as humans [5,8,9,10].

In the last 30 years, an ever-increasing number of people have employed natural remedies to maintain good health as well as to treat several diseases [11,12], and there has been growing interest in *Citrus* derivatives as weapons to fight different chronic illnesses [13,14]. In this line, several natural products used as nutraceuticals or dietary supplements are taken to combat excessive adiposity and promote weight loss [15,16,17], including *Citrus* extracts [18,19]. Their antiadipogenic and antiobesity activities are mainly due to the pool of polyphenols, especially the flavonoids, that are present in these extracts [20]. Indeed, numerous studies performed both in vitro and in vivo have demonstrated that several dietary flavonoids can modulate molecular mechanisms involved in lipid metabolism, thus promoting a role in treating adiposity, obesity, and their associated metabolic diseases [20,21]. In particular, the ability of *Citrus* juices and their components to induce lipolysis, reduce fat accumulation, or regulate enzymes related to obesity in several experimental models has been reviewed by Rampersaud and Valim [22]. *Citrus sinensis* is the most well-known *Citrus* worldwide, and its juice (OJ) is commonly consumed for both its taste and beneficial properties [23,24,25]. The variety Tarocco, cultivated especially in eastern Sicily (Italy) and commercially called “half-blood” orange, has been positively associated with several nutritional, dietary, and health benefits [26]. Titta et al. [27] showed that OJ diminished high-fat diet-induced body weight gain in mice, reducing the fat mass in both abdominal and inguinal regions by 50%. The histological examination of the adipose tissue displayed a marked reduction in the size of the adipocyte cells and lipid accumulation in OJ-treated mice compared with untreated ones [27]. Moreover, some studies performed with humans found anti-obesity activity in OJ consumers that, however, was not found in other ones. O’Neil et al. [23] reported a 21% reduced risk of obesity in adults that consumed 100% OJ compared with non-consumers. The intake of OJ, either normal or highly concentrated in polyphenols, counteracted the increase of body weight in overweight or obese adults and modified several antioxidant enzymes, thus protecting them against DNA damage and lipid peroxidation [28]. Furthermore, the results of a clinical trial carried out in overweight human healthy volunteers to evaluate the effects of a commercial Moro OJ extract on decreasing body weight showed a significant reduction in waist and hip circumference in subjects supplemented with the OJ extract in comparison with nontreated ones [29]. Conversely, Azzini et al. [30] did not find any significant effect on body weight in women taking 500 mL daily of commercial OJ; also, 100% OJ did not affect weight loss induced by a reduced-calorie diet, although it provided other health benefits [31]. These conflicting results have left some doubt regarding the real anti-obesity capacity of OJ and its derivatives, suggesting the need to deepen the studies aimed at examining their role in reducing adiposity. Based on these observations, this study was designed to evaluate the effect of a flavonoid-rich extract of Tarocco OJ (OJe) on diet-induced obese zebrafish, investigating its mechanism of action.

## 2. Results

### 2.1. OJe Reduced Both Normalized Body Weight (BW) and Body Mass Index (BMI) in Both Normally Fed (NF) and Overfed (OF) Zebrafish

**Figure 1:**
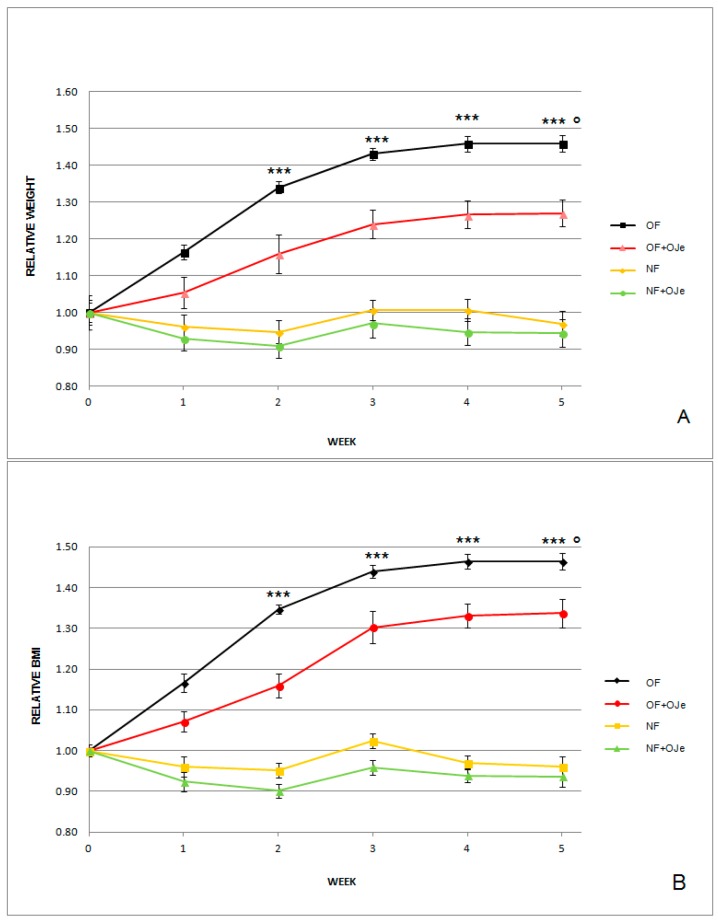
Effects of the orange juice extract (OJe) on both normalized body weight (BW) and body mass index (BMI) in overfed (OF) and normally fed (NF) zebrafish. Graphs show the fold change of BW (**A**) and BMI (**B**) values recorded each week during the five experimental weeks. Results are expressed as mean ± SD of data collected from 15 animals per experimental group. BW and BMI relative values were extrapolated from the data detected in the fish at T_0_ for each group, which were arbitrarily expressed as 1. At T_0_, the BW values were the following (g): 0.44 in OF, 0.49 in OF supplemented with OJe (OF + OJe), 0.54 in NF, and 0.45 in NF supplemented with OJe (NF + OJe) groups. The BMI values (g/cm^2^) at T_0_ were 0.027 and 0.030 in the OF and OF + OJe groups, respectively, while they were 0.031 and 0.030 in the NF and NF + OJe groups. *** *p* < 0.001 vs. OF + OJe; ° *p* < 0.001 vs. NF.

As expected, at the end of the second, third, fourth, and fifth week of the experiment, the group of OF zebrafish had significant gains (*p* < 0.001) in terms of both BW and BMI with respect to the fish fed with the equivalent of 20 mg *Artemia* cysts/day (NF) (Figure 1).

In particular, as shown in Figure 1A, after five weeks from the start of the experiments, the BW increased in both OJe-treated and untreated OF groups, although OF (1.4640 ± 0.0059) displayed a greater BW increase than OF supplemented with OJe (OF + OJe) (1.2696 ± 0.0359; *p* < 0.001). However, BW showed statistically significant differences between OF vs. OF + OJe starting from the end of the second week (*p* < 0.001). Moreover, starting from the first week of the experiment, compared with the matched untreated group (NF), the NF treated with OJe (NF + OJe) showed a modest decrease in BW.

The BMI showed the same trend observed for the BW. Indeed, after the second week of treatment, in the OF + OJe group, we observed a significant reduction (*p* < 0.001) of this index with respect to the OJe-untreated one (Figure 1B). At the end of the fifth week, the major values (Figure 1B) were reported in the OF group that did not receive OJe (OF = 1.4640 ± 0.0100), which differed statistically (*p* < 0.001) from those found in the group of fish supplemented with OJe (OF + OJe = 1.3374 ± 0.0340). Conversely, in the OJe-treated and untreated NF fish, we found a negligible decrease in BW. However, we did not observe any sign of OJe-induced toxicity, as suggested by their normal behavior and the fact that all zebrafish involved in this study ate the whole dose of food given. This indicates both the safety of this dose of OJe and that the reduction in BW and BMI observed in OF + OJe group was not due to a reduction in eating *Artemia*.

### 2.2. OJe Decreased Adipose Tissue in Overfed Zebrafish

**Figure 2:**
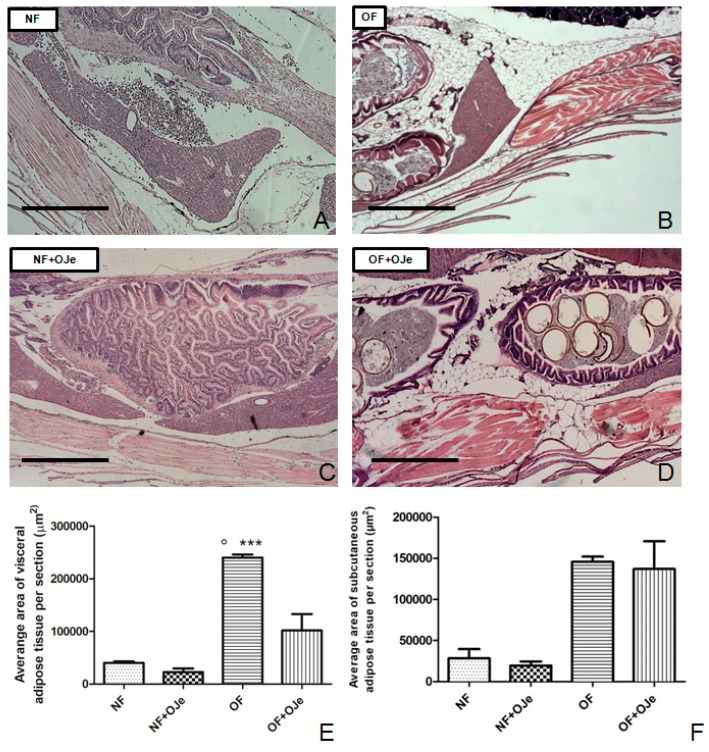
OJe reduced the development of adipose tissue in overfed zebrafish. Histological examination of abdominal tissue of sagittal sections, performed through hematoxylin and eosin staining, showed the rise of visceral (**E**) and subcutaneous (**F**) fat depots in the OF group (**B**) but not in the NF group (**A**). Five weeks of OJe treatment reduced visceral adipose tissue area in OF fish (**D**,**E**) without affecting the subcutaneous area (**D**,**F**). The morphometric fat analysis showed that NF fish had no significant changes in either visceral (**C**,**E**) or subcutaneous (**C**,**F**) adipose tissue areas. Photos (**A**–**C**) are representative of that (three per section) taken in 10 fish per group. The values in graphs (**E**) and (**F**) are expressed in µm^2^ of adipose tissue per section. Scale bars correspond to 1 mm. Data presented in the graphs are the mean ± SD of 10 fish per each group. *** *p* < 0.001 vs. respective counterpart; ° *p* < 0.001 vs. NF.

After five weeks of treatment, the morphometric analysis of adipose tissue showed a different development among the four groups. Indeed, we found a significant difference in the average area of both visceral and subcutaneous adipose tissue between NF and OF (*p* < 0.001), indicating that a triplicated daily dose of food intake determines the growth of an abundant layer of adipose tissue, likewise with humans. Interestingly, at the visceral level, the average area of adipose tissue in the OF + OJe group (101,893 ± 31,320; Figure 2D,E) decreased significantly (*p* < 0.001) in comparison with the OF group (240,413 ± 6056; Figure 2B,E) but not at the subcutaneous level (*p* = 0.9867; Figure 2F). Conversely, OJe did not produce any significant effect either at the subcutaneous (Figure 2A,C,F) or at the visceral (Figure 2A,C,E) levels of NF fish.

**Figure 3:**
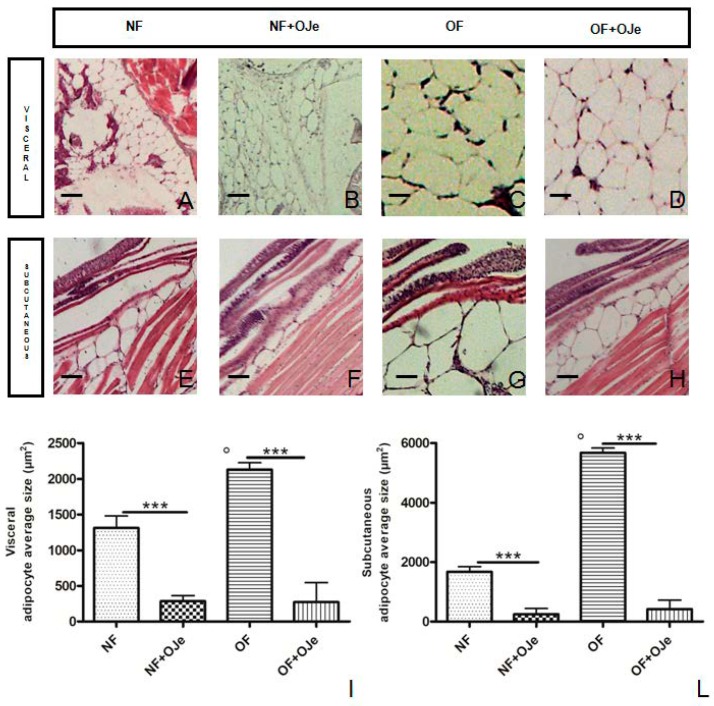
Effect of OJe on visceral and subcutaneous adipocyte size. Histological features of abdominal adipose tissue in sagittal sections (hematoxylin and eosin staining) showing visceral (**A**–**D**,**I**) and subcutaneous (**E**–**H**,**L**) adipocyte size in the four experimental groups. OJe treatment significantly reduced both visceral (**A**–**D**,**I**) and subcutaneous (**E**–**H**,**L**) average adipocyte size in both NF and OF fish. Photos (**A**–**H**) are representative of that (three per section) taken in 10 fish per group. Bar graphs show the results of the morphometric analysis of fat performed in both visceral (**I**) and subcutaneous (**L**) adipocyte average size in the four experimental groups (NF, NF + OJe, OF, and OF + OJe). Scale bars correspond to 20 µm. Results from the morphometric analysis in graphs (**I**) and (**L**) are expressed in µm^2^ of adipocyte size and expressed as mean ± SD of 15 fish per each group. *** *p* < 0.001 vs. respective counterparts; ° *p* < 0.001 vs. NF.

Moreover, the average size of visceral adipocytes differed significantly (*p* < 0.001; Figure 3A–D, I) between NF (1312.00 ± 11.44; Figure 3A,I) and NF + OJe (284.50 ± 40.30; Figure 3B,I) groups, as well as between OF (2128.00± 6.53; Figure 3C,I) and OF + OJe (271.20 ± 18.77; Figure 3D,I) groups, indicating a possible reduction in cell size. Conversely, the average size of subcutaneous adipocytes showed a significant difference (*p* < 0.001; Figure 3E–H,L) between NF (1680.00 ± 12.01; Figure 3E,L) and OF (5677.00 ± 11.48; Figure 3G,L), NF versus NF + OJe (248.70 ± 13.25; Figure 3F,L), and OF versus OF + OJe (416.80 ± 21.58; Figure 3H,L).

**Figure 4:**
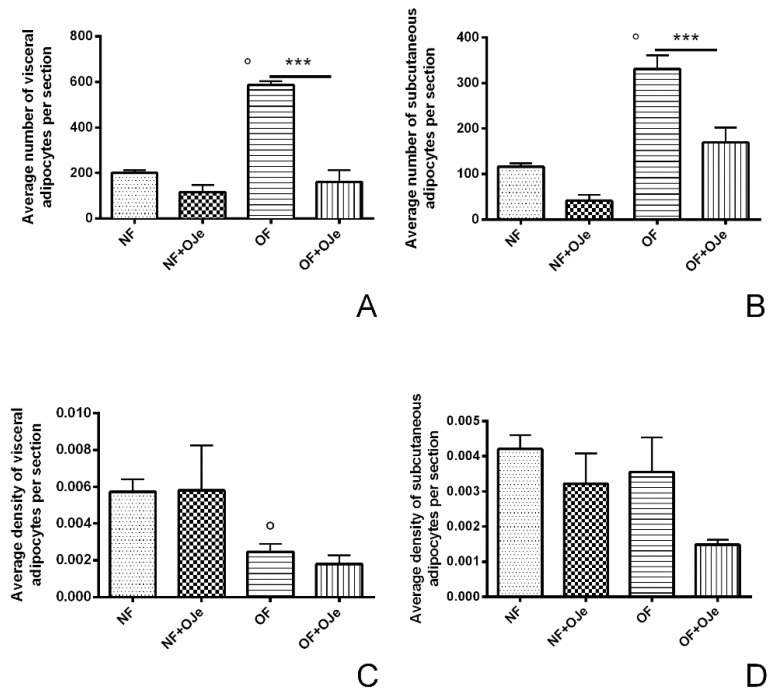
OJe reduced adipocyte number and density in OF zebrafish. Morphometric analysis of visceral (**A**) and subcutaneous (**B**) fat displays a reduction in the number (**A**,**B**) of adipocytes in OF + OJe fish compared with OF untreated ones, while their density was statistically reduced only at the subcutaneous level (**D**) but not at visceral one (**C**). Values reported in the graphs are expressed in µm^2^ of adipose tissue per section and represent the mean ± SD of 10 animals per each group. *** *p* < 0.001 vs. respective counterparts; ° *p* < 0.001 vs. NF.

Five weeks of excessive feeding significantly increased the number of visceral adipocytes (OF vs. NF, *p* < 0.001), while the treatment with OJe significantly reduced this marker of obesity (OF, 586.70 ± 17.08 and OF + OJe, 160.20 ± 51.99; *p* < 0.001; Figure 4A), thus suggesting the great lipolytic action of OJe (see also Figure 3I). Instead, in the NF group, this effect did not reach statistical significance (NF, 200.00 ± 12.44 and NF + OJe 116.10 ± 31.18; *p* = 0.2557; Figure 4A). No difference between the OJe-treated and -untreated groups was recorded in the density of visceral adipocytes (Figure 4C). OJe also reduced the number of subcutaneous adipocytes in both OF and NF fish, however, reaching statistical significance only between the OJe-treated and -untreated OF animals (OF 330 ± 5.6 and OF + OJe 169 ± 33; *p* < 0.001; Figure 4B).

On the contrary, we did not find differences in the density of visceral adipocytes (Figure 4C) or in the subcutaneous adipocytes of NF groups (Figure 4D), but it was significantly reduced in the OF + OJe group in comparison with the matched one (*p* < 0.001; Figure 4D).

### 2.3. Gene Expression Analysis Following Citrus Extract Administration

In order to assess the mechanism through which OJe induced its anti-obesity effect, quantitative PCR (qPCR) analysis was carried out on brain and gut samples from the four experimental groups. In particular, the expression of orexigenic (ghrelin, orexin, and neuropeptide Y (NPY)) and anorexigenic (leptin A and pro-opiomelanocortin (POMC)) genes were assessed. Indeed, as in mammals, also in fish, the regulation of food intake is a complex process carried out by several mechanisms in both peripheral locations and the brain, especially in the hypothalamus [32].

**Figure 5:**
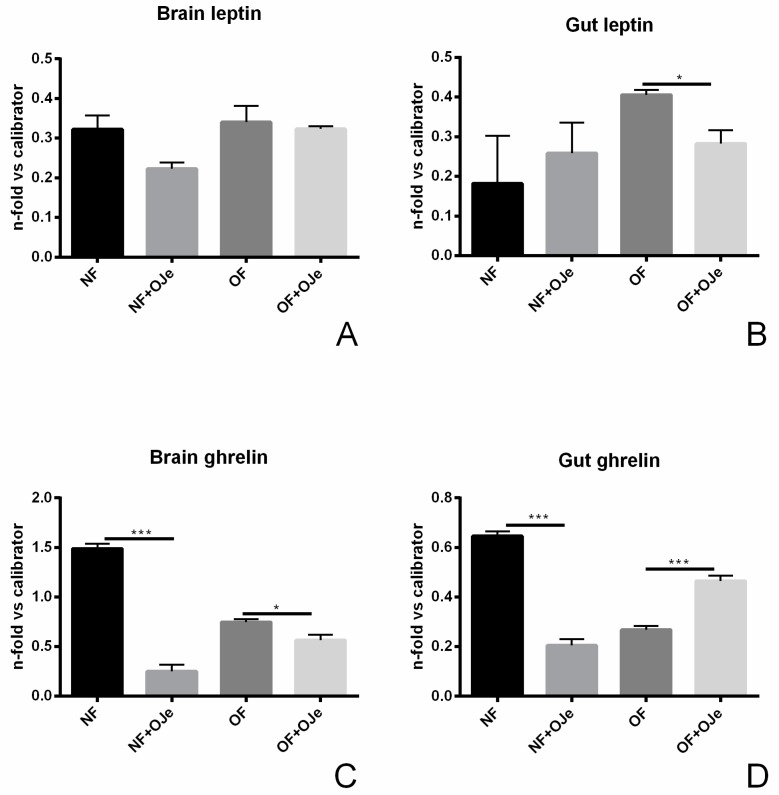
Effects of OJe on the expression of obesity-related genes. The expression of leptin A (**A**,**B**) and ghrelin (**C**,**D**) was evaluated by qPCR in both brains (**A**,**C**) and guts (**B**,**D**). Relative quantities of mRNA were calculated using the 2^−ΔΔ*C*t^ quantification method. Results are expressed as fold change in OJe-treated fish compared to untreated ones, after normalization to β-actin. Data represent the mean ± SD of five animals for each group. * *p* < 0.05, ** *p* < 0.01, *** *p* < 0.001 vs. respective counterparts.

Leptin and ghrelin are two peripheral hormones recognized for their major role in energy balance. The former is recognized as a mediator of long-term regulation of energy balance and a suppressor of food intake, thus causing weight loss, while ghrelin is a fast-acting hormone implicated in meal initiation. As shown in Figure 5A, in the brain, we did not find any significant difference between the level of leptin A gene expression in the four experimental groups, although in NF, a reduction ascribable to OJe was noticed. Instead, in the gut of OF fish treated with the extract, we observed a significant reduction of the leptin A level (*p* < 0.05; Figure 5B). Analysis of ghrelin mRNA expression in the brain (Figure 5C) showed that OJe was able to greatly reduce its level in NF zebrafish tissues (*p* < 0.001 vs. not supplemented NF fish), which, albeit to a minor extent, was also found in the OF groups (*p* < 0.05). Moreover, we observed that ghrelin gene expression in the gut of NF fish treated with OJe was more than halved with respect to the untreated ones, while in OF fish supplemented with OJe, its level was nearly 2-fold higher than in untreated ones (*p* < 0.001 in both cases; Figure 5D).

**Figure 6:**
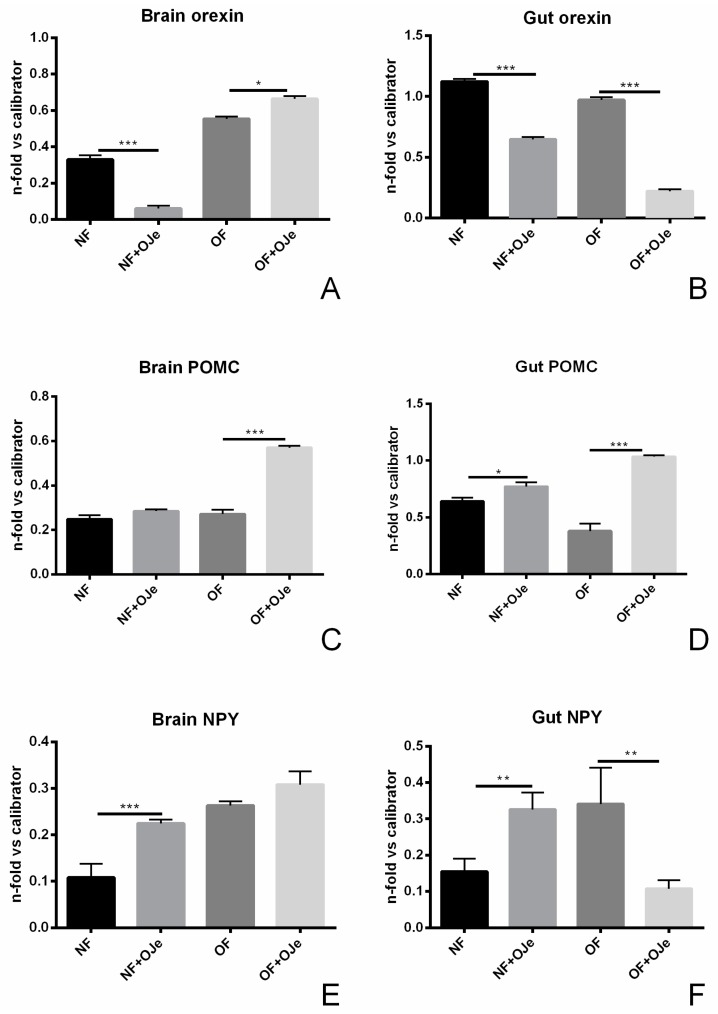
OJe is capable of modulating the expression of orexin, pro-opiomelanocortin (POMC), and neuropeptide Y (NPY). Brain (**A**,**C**,**E**) and gut (**B**,**D**,**F**) tissues from OJe-treated and -untreated fish were processed for qPCR analyses in order to investigate orexin (**A**,**B**), POMC (**C**,**D**), and NPY (**E**,**F**) gene expression. The levels of mRNA were calculated by the 2^−ΔΔ*C*t^ relative quantification method. Results are expressed as fold change in OJe-treated fish compared to those found in untreated ones, after normalization to β-actin. Data represent mean ± SD of five animals per each group. * *p* < 0.05, ** *p* < 0.01, *** *p* < 0.001 vs. respective counterparts.

Orexin, also known as hypocretin, is a neurotransmitter that plays a relevant role in the regulation of the sleep–wakefulness cycle and appetite. In this frame, it acts in the control of food intake, metabolism, and overall energy balance because it stimulates food-seeking behavior promoting food intake, as well as increases energy expenditure [33]. In our study, five weeks of treatment with OJe significantly decreased the orexin mRNA level both in the brain and gut of NF fish (*p* < 0.001 vs. untreated fish), as well as in in the gut of OF animals (*p* < 0.001; Figure 6B), while it raised the level of the neurotransmitter in the brain (*p* < 0.05; Figure 6A).

POMC is a neuropeptide produced by neurons present in the hypothalamic arcuate nucleus and is cleaved to provide peptides involved in several functions, including appetite and food intake control; its production results from the integration of information on nutrients and hormones levels, as well as circadian signals [32]. Its gene level was increased in both the brain and gut of OF fish supplemented with OJe (Figure 6C,D, respectively; *p* < 0.001 vs. untreated animals), while in NF zebrafish, we found a small but significant increase of POMC expression only in the gut (*p* < 0.05; Figure 6D).

NPY is a key neurotransmitter involved in the control of energy balance, including stimulation of food intake and inhibition of energy expenditure [32]. Figure 6 shows the effect of OJe on brain and gut NPY expression. Its level was raised significantly in both tissues of NF zebrafish (*p* < 0.001 in the brain, Figure 6E; *p* < 0.01 in the gut, Figure 6F), whereas, it was diminished in the gut of OF (*p* < 0.01; Figure 6F), without significant differences in the brain (Figure 6E).

## 3. Discussion

The health-promoting properties of OJ are well known. They are linked to a nonspecific antioxidant effect, as well as the ability of its biomolecules to modulate many antioxidant systems involved in the etiology of numerous oxidative-based diseases. Moreover, OJ as well as *Citrus* polyphenols could help managing obesity and its related chronic diseases [34].

Recently, we assessed the antioxidant capability of a flavonoid-rich extract of Tarocco OJ (OJe), which showed its ability to reduce both reactive oxygen species and membrane lipid peroxidation, improving mitochondrial functionality and preventing DNA oxidative damage in A549 cells stressed by H_2_O_2_ [35]. OJe also showed its chelating property and its capability to induce the catalase enzyme, thus stopping the iron oxidative-induced cascade [36]. Finally, OJe demonstrated anti-inflammatory and neuroprotective effects in vivo by modulating specific signaling pathways involved in degenerative processes [37,38].

In this study, we demonstrated that the pool of flavonoids in OJe exerts an anti-obesity effect on fat accumulation in diet-induced obese zebrafish. We had already employed this experimental model, which shares several pathophysiological features with mammalians, in order to study the mechanisms underlying obesity [8,9,10].

**Figure 7:**
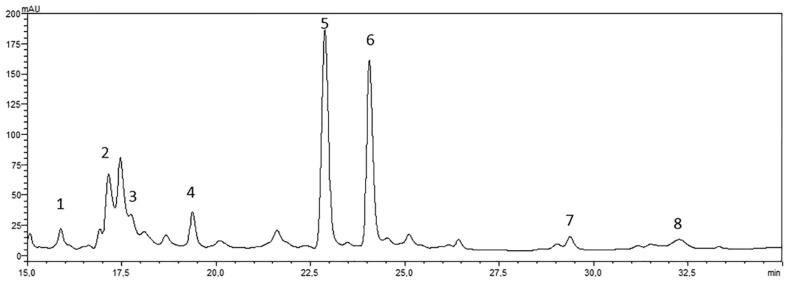
Reversed-phase high-performance liquid chromatography coupled with diode array detection (RP-HPLC-DAD) separation of flavonoids present in OJe. UV–Vis spectrum of the eluted molecules was monitored between 200 and 800 nm. The chromatogram was recorded at 278 nm. Flavonoids corresponding to peaks 1–7, expressed in milligrams (mg) per liter (L) of aqueous extract (mg/L), are the following: (1) lucenin-2 (6.2); (2) vicenin-2 (16.1); (3) lucenin-2-4′-methyl ester (9.5); (4) eriocitrin (11.9); (5) narirutin (62.8); (6) hesperidin (56.4); (7) sinensetin (0.52); (8) nobiletin (5.29).

First, we assessed that OJe did not alter the intake of food by NF or OF fish by checking that they ate the whole dose of *Artemia* nauplii given. This suggests that the zebrafish reduced weight gain induced by OJe was not caused by a possible inhibition in food intake, but rather by events occurring after the ingestion of food. Moreover, some studies suggested that OJ is able to reduce increases in weight [23,28,29], indicating that multiple components present in OJ might act synergistically to inhibit fat accumulation [27], yet without indicating which active compound was able to exert this beneficial effect. In this experimental research, we employed a well-characterized OJe that clearly indicates its flavonoids as being responsible for the anti-obesity and lipolytic activity of OJ. It contains high amounts of hesperidin, followed by narirutin, vicenin-2, nobiletin, and lucenin-2 methyl ether (Figure 7), as shown previously [35,37]. Interestingly, the antiadipogenic and delipidating effect of hesperidin has been reported in both in vitro [39] and in vivo [40] models. Similarly, nobiletin decreased both white adipose tissue and body weight gain in high-fat diet-induced obese mice by regulating the expression of lipid-metabolism-related and adipokine genes, as well as that of inflammatory markers and the activity of the insulin signaling pathway [41]. These findings suggest that, at least in part, both hesperidin and nobiletin can be responsible for the reduction of weight gain given by OJe.

It is undoubtedly acknowledged that an increased energy intake for a long period of time without being compensated by an increase in energy consumption promotes the growth of adipose tissue mass. This occurs through the augment of both adipocyte number (hyperplasia) and/or size (hypertrophy). The latter leads to the former, which is generally linked to the severity of obesity [42]. Therefore, inhibition of adipocyte hypertrophy and hyperplasia can be exploited to prevent adipogenesis and, hence, obesity. Nowadays, the latter represents one of the most prevailing health concerns worldwide, with the incidence increasing at a high rate, resulting in enormous social costs. Currently available drugs for weight management include substances able to increase metabolism or reduce appetite or fat absorption, such as stimulants of the central nervous system or peripherally acting anti-obesity drugs. Unfortunately, they are associated with serious side effects such as anxiety, palpitations, insomnia, hyperthyroidism, and gastrointestinal disturbances, thus encouraging the search of safer anti-obesity agents. Several studies have shown the potential of natural products to counteract weight gain, especially due to the effects of flavonoids on adipogenesis [43]. In this context, here, we show that, in overfed zebrafish, OJe decreased the visceral adipose tissue without any effect at the subcutaneous level. Interestingly, OJe did not affect the adipose layer in the NF fish, suggesting its possible ability to counteract the increase in adipose tissue in the abdominal area, which is the most affected in overweight subjects. This OJe-induced contraction of visceral adipose tissue observed in the OF zebrafish, at least in part, was due to the reduction in both adipocyte number and size, thus suggesting the lipolytic capability of OJe.

A major role in the increase/decrease of both fat and body weight is played by gut hormones that can act on the central nervous system (CNS), particularly in the hypothalamus, thus leading to orexigenic/anorexigenic effects [44]. It has been shown that flavonoids can modulate mRNA and/or protein synthesis of several obesity-related genes [45]. Among these, leptin and ghrelin are known to be keystones of energy balance, to the extent that they can be considered targets for treating obesity. Leptin is a fundamental peripheral hormone that, increasing after each meal, induces the feeling of satiety in the brain; its expression and secretion rise in obesity. In normal conditions, when the mature adipocytes accumulate triglycerides, they secrete several adipokines, including leptin, which reduces food cravings. Conversely, in the obese, the abnormal accumulation of triglycerides in adipocytes makes them hypertrophic, leading to an overexpression of leptin and, consequently, “leptin resistance” [46]. Moreover, in these conditions, cells secrete proinflammatory mediators, which in turn increase adipokine production. Of note, in our experiments, we observed a decrease of the leptin A level in the gut of OF zebrafish, which was likely due to the reduction of adipocytes. Indeed, this peptide is mainly expressed in mammalian adipose tissue, although in fish and birds, it is produced in both hepatocytes and, in smaller amounts, adipocytes. Ghrelin is a peripheral orexigenic hormone produced mainly by the stomach that plays a physiological role in both meal initiation and the ingestion of food. It is upregulated in conditions of malnutrition, such as anorexia, and is downregulated in positive energy balance states, such as obesity. The pre-prandial surge of plasma ghrelin stimulates food intake, while its levels decrease after consumption of a meal. Moreover, it induces both an increment of adiposity gains and caloric storage [47]. On the other hand, hesperidin, one of the main components of OJe, was shown to potentiate ghrelin signaling [48], reinforcing our hypothesis. Orexin, also known as hypocretin, is a neuropeptide synthesized by a small number of neurons in the dorsolateral hypothalamus and perifornical area. It has been demonstrated that orexin exerts a multitasking role in health and disease, since it regulates vital body functions, including the sleep/wake cycle, feeding behavior, energy homeostasis, reward systems, cognition, and mood [32,33]. Orexin acts both centrally and peripherally to affect energy expenditure and fat storage, leading to negative energy balance and reduced adiposity. In our experiments, we observed that OJe significantly increased the orexin level in the brain of OF zebrafish, in line with what has been found in experimental models, as reviewed by Teske and Mavanji [49]. Our results are in accordance with those reported by Nakamachi et al. [50], who claimed that high hypothalamic orexin levels are associated with raised locomotor activity. This occurred also in mice, where this neuropeptide was linked to an increase in spontaneous physical activity, accompanied by an increase in energy expenditure without changes in overall chow intake [51]. Indeed, orexin activation in OF zebrafish may have raised the metabolic rate, thus inducing weight and adiposity loss. With respect to OF fish, data regarding orexin found in NF suggest that OJe can act differently depending on the physio-pathological condition. The regulation of food intake and energy expenditure is also fine-tuned by the neuropeptides POMC and NPY, which, together with hormones and circadian signals, participate in the control of energy balance both in mammals and fish [32]. The endogenous POMC tone, inhibiting feeding and energy storage, may vary according to fat intake or energy balance. Administration of OJe increased POMC expression in OF fish, both centrally and peripherally, suggesting its role in the energy expenditure that may have contributed to the reduction of adiposity and weight gain observed in OJe-supplemented OF fish. Indeed, POMC is the precursor of signaling peptides known as melanocortins, the receptors of which are spread throughout the body and are involved in the regulation of energy balance as well as appetite control. Their action in brown adipose tissue increases thermogenesis, thus stimulating weight loss [52], while in the hypothalamus, they inhibit NPY neurons, thus leading to an anorexigenic effect. NPY is the most potent orexigenic neuropeptide in vertebrates, although findings regarding its mRNA abundance in zebrafish are contradictory [32]. In our experiments, as expected, diet-induced obesity led to increased NPY expression both centrally and peripherally, supporting its role in obesity. The significant reduction of NPY expression in the gut of OF fish supplemented with OJe may have increased the metabolic rate through reinforcing the peripheral effect of melanocortins, since NPY is an endogenous antagonist of their receptors. Therefore, our data indicate that the anti-obesity effect of OJe may be linked to its ability to modulate the metabolism-regulating hormones of the brain-gut axis.

The link between inflammation and obesity is well known [53]. On the other hand, obesity can be considered chronic low-grade inflammation of the adipose tissue, causing increased generation of reactive species, adipokines, and other proinflammatory factors that are interconnected with fat gain, thus feeding a vicious cycle. Therefore, anti-inflammatory drugs can interrupt this dangerous chain, thus helping to manage both obesity and its related comorbidities. Of note, *Citrus* juices and their derivatives have been shown to possess antioxidant and anti-inflammatory activities [54,55,56,57]. In this line, we recently assessed the antioxidant capability of OJe, showing that it reduced both reactive oxygen species and membrane lipid peroxidation, improving mitochondrial functionality and preventing DNA oxidative damage in A549 cells stressed by H_2_O_2_ [35]. OJe also exhibited a chelating property and the ability to induce the catalase enzyme, thus stopping the iron oxidative-induced cascade [36]. Finally, OJe demonstrated anti-inflammatory and neuroprotective effects in vivo by modulating specific signaling pathways involved in degenerative processes [35,36,37,38]. These findings support the hypothesis that the anti-inflammatory activity of OJe supports the effect of this extract against fat gain.

This is the first study that focused on the anti-obesity effect exerted by the pool of flavonoids in OJe on fat accumulation in diet-induced obese zebrafish, achieved through a lipolytic action linked to a restoration of metabolism-regulating gene expression, strengthening their role as weight management agents.

## 4. Materials and Methods

### 4.1. Ethics Statement

The experimental procedures employed in this study were in accordance with the principles outlined in the Declaration of Helsinki and with the Italian law regarding the care and use of laboratory animals (National Law n. 26/2014). The Italian Ministry of Health approved the experimental protocol (A.M. n.50, 8 August 2013).

### 4.2. Drug

The OJe was provided by the company Agrumaria Corleone (Palermo, Italy). The fruits of *Citrus sinensis* (L.) Osbeck (sweet orange) var. Tarocco were from crops situated in the southeast of Sicily, Italy. The extract was obtained in its liquid form by passing the fresh OJ in columns containing adsorbent resins that restrain flavonoids. These were eluted with NaOH and then acidified to avoid flavonoid modification. The aqueous OJe was stored at −20 °C until use. The study was carried out employing the same OJe already used in other studies [35,36,37]. Before starting the experiments, we repeated the reversed-phase high-performance liquid chromatography coupled with diode array detection (RP-HPLC-DAD) analysis of flavonoids present in OJe (Figure 7). Their identification was achieved by comparing their spectra and retention times with those of commercial standards (Extrasynthese, Genay, France), as described in [58]. The results of the qualitative and quantitative assays reflect those reported previously [35,37].

### 4.3. Zebrafish Husbandry, Experimental Groups, BW and BMI Measurements, and Tissue Preparation

Adult male zebrafish (*n* = 60, 12-months old) were obtained from a breeding colony at C.I.S.S. (Centre of Experimental Ichthyopathology of Sicily, Department of Veterinary Science, University of Messina), kept at a constant temperature of 28.5 °C, and fed once a day. The fish were randomly divided into four dietary groups (*n* = 15 fish each) as follows: (i) normally fed (NF); (ii) overfed (OF); (iii) NF supplemented with OJe (5 mL/L in fish water; NF + OJe); and (iv) OF supplemented with OJe (OF + OJe). Each week, BW and BMI were measured, and at the end of the fifth week, euthanized zebrafish were processed for both microscopic evaluations and qPCR analyses:

(1) NF: animals fed with the equivalent of 20 mg cysts/fish/day of freshly hatched *Artemia* nauplii; 

(2) OF: fish fed with the equivalent of 60 mg cysts/fish/day (20 mg cysts/fish three times a day) of freshly hatched *Artemia* nauplii;

(3) NF + OJe: fish living in 5 mL of OJe per liter of water, fed with the equivalent of 20 mg cysts/fish/day of freshly hatched *Artemia* nauplii;

(4) OF + OJe: fish living in 5 mL of OJe per liter of water, fed the equivalent of 60 mg cysts/fish/day of freshly hatched *Artemia* nauplii.

After feeding, we observed the fish until their dose was thoroughly eaten, as well as randomly checked them in order to assess any possible sign of change in behavior throughout the day. The entire protocol took five weeks to complete. In this study, zebrafish were separated in different tanks (one zebrafish per 1 L tank, according to Oka et al. [5] and Montalbano and colleagues [9,10]). Water with or without OJe was changed every day. Throughout the course of treatment, in accordance with the protocol of Montalbano et al. [9], both the BW and the length of every fish were measured each week in order to evaluate the increase in BMI levels. After five weeks of treatment, the fish were fasted overnight and then euthanized with a lethal dose of MS 222 (ethyl 3-aminobenzoate methanesulfonate, 0.2 g/L; Sigma, Saint Louis, MO, USA). Afterwards, brains and intestines from five fish per group were quickly removed and processed in order to isolate the RNA for qPCR. The remaining fish (*n* = 10 fish per group) were fixed in Bouin’s fixative and routinely processed for light microscopy.

### 4.4. Gene Expression of Leptin, Ghrelin, Orexin, POMC, and NPY

Total RNA was extracted from whole zebrafish brains and guts using the TRIzol reagent (Invitrogen, Carlsbad, CA, USA); then, 2 μg of total RNA was reverse transcribed into cDNA using the High-Capacity cDNA Archive Kit (Applied Biosystems, Foster City, CA, USA), as previously described [59,60]. The mRNA levels of leptin A, ghrelin, orexin, POMC, and NPY expression were analyzed by qPCR using a TaqMan^®^ universal PCR Master Mix (Applied Biosystems) [10]. The primers were designed based on the published zebrafish mRNA sequences for the genes analyzed. Table 1 lists the GenBank accession numbers and primer sequences. The assays were performed in triplicate using a 7500 PCR real-time system (Applied Biosystems). The results were calculated using the 2^−ΔΔ*C*t^ algorithm against β-actin and expressed as the *n*-fold difference compared to an arbitrary calibrator, chosen as a higher value than ΔΔ*C*_t_s.

### 4.5. Analysis of Zebrafish Fat Tissue and Adipocytes

The morphological studies of zebrafish were carried out on histological sections according to the procedure by Montalbano and colleagues [9,10].

### 4.6. Statistical Analysis

The assays were carried out in triplicate. All experimental data are reported as mean ± SD. Statistical analyses of BW and BMI values were performed by two-way repeated measures ANOVA. *p*-values lower than 0.05 were considered significant. Statistical analyses of gene expression and fat morphometry were performed by standard two-way ANOVA, and any significant difference was determined at a significance level of 0.05 via the application of a Tukey’s test. *p*-values lower than 0.05 were considered significant. For all statistical analyses, we used GraphPAD software.

## Figures and Tables

**Table 1:** Oligonucleotide primers used for real-time PCR.

Gene Product	GenBank Accession Number	Primer Sequence
Leptin A	NM_001128576	Forward: 5′-CATCATCGTCAGAATCAGGG-3′ Reverse: 5′-ATCTCGGCGTATCTGGTCAA-3′
Ghrelin	EU908735.1	Forward: 5′-CAAGAGTGGGCAGAAGAGAA-3′ Reverse: 5′-ATGTAGTTGTAGTGGATGGT-3′
Orexin	NM_001077392.2	Forward: 5′-GCTCCTTGCAAACTACGAG-3′ Reverse: 5′-GAGTTGTGCAGCAGCAGTTG-3′
POMC	AY125332.2	Forward: 5′-TGAACAGATAGAGCCGGAGT-3′ Reverse: 5′-ACCTCGTTATTTGCCAGTC-3′
NPY	BC162071	Forward: 5′-TGAAGATGTGGATGAGCTGG-3′ Reverse: 5′-CACCATGCCAAATGATCCTC-3′
β-Actin	NM_131031	Forward: 5′-TTGCCCCGAGGCTCTCTT-3′ Reverse: 5′-AGTTGAAGGTGGTCTCGTGGAT-3′

## Abbreviations

BMI	Body mass index
BW	Body weight
OF	Overfed
NF	Normally fed
OJe	Orange juice extract
POMC	Pro-opiomelanocortin
NPY	Neuropeptide Y
